# Supplementary material for: Prolonged hospitalization signature and early antibiotic effects on the nasopharyngeal resistome in preterm infants
Source: Nat Commun. 2024 Jul 17;15:6024. doi: 10.1038/s41467-024-50433-7 (PMC11255206; doi:10.1038/s41467-024-50433-7)
Supplement: Supplementary file 1 — Supplementary Information [file 41467_2024_50433_MOESM1_ESM.pdf]

Prolonged hospitalization signature and early antibiotic effects on the nasopharyngeal resistome in preterm infants

Achal Dhariwal[1]†, Polona Rajar[1,2]†, Gabriela Salvadori[1], Heidi Aarø Åmdal[1], Dag Berild[3,4], Ola Didrik Saugstad[5], Drude Fugelseth[2,4], Gorm Greisen[6], Ulf Dahle[7], Kirsti Haaland[2], Fernanda Cristina Petersen[1]*

†These authors contributed equally

*Corresponding Author: Fernanda Cristina Petersen (f.c.petersen@odont.uio.no)

Supplementary Information

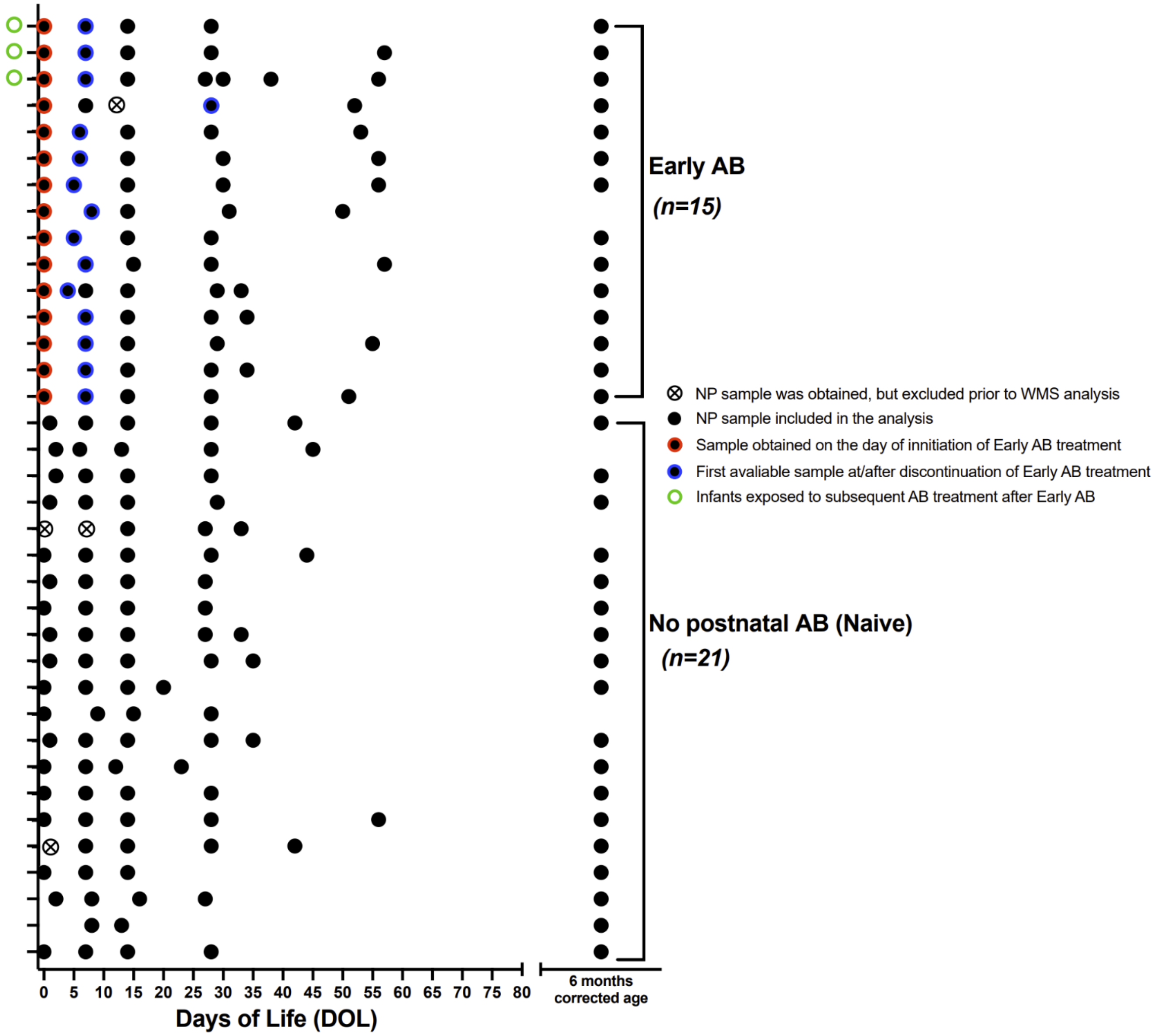

Supplementary Fig. 1: Sample collection and antibiotic exposure for preterm infants in this study. Sampling timeline of 198 nasopharyngeal samples collected from 36 preterm infants over first six months (corrected age), together with information regarding the time of early antibiotic exposure (red and blue colored circles), exposure to subsequent antibiotic treatment (green colored circles) and included/excluded samples. The x-axis represents the days of life (i.e., corrected age) and the y-axis represents the infants. NP = nasopharyngeal; AB = antibiotics.

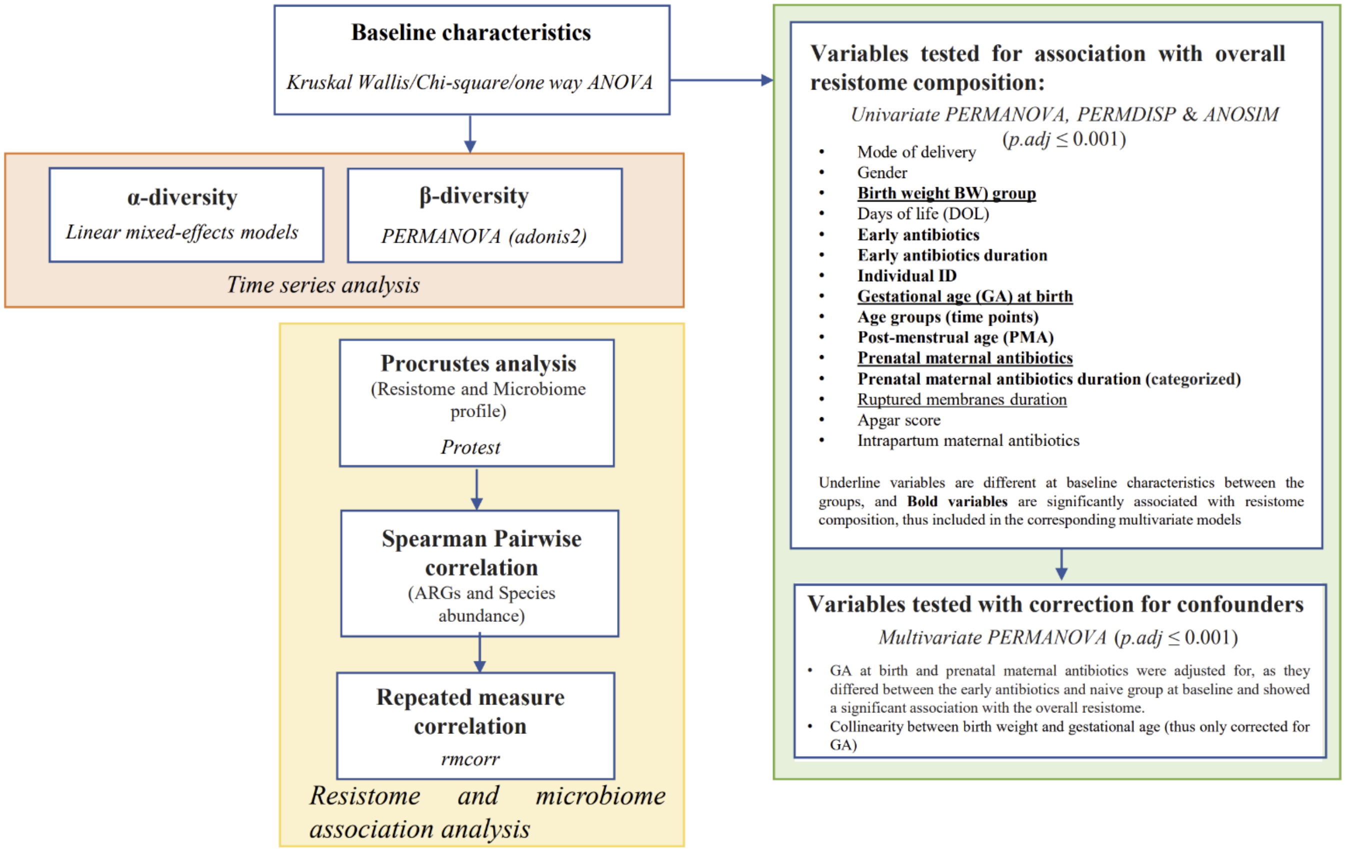

Supplementary Fig. 2: Statistical analysis design used in the study. ARGs = antibiotic resistance genes; PERMANOVA = permutational multivariate analysis of variance; PERMDISP = permutational analysis of multivariate dispersion; ANOSIM = analysis of similarities.

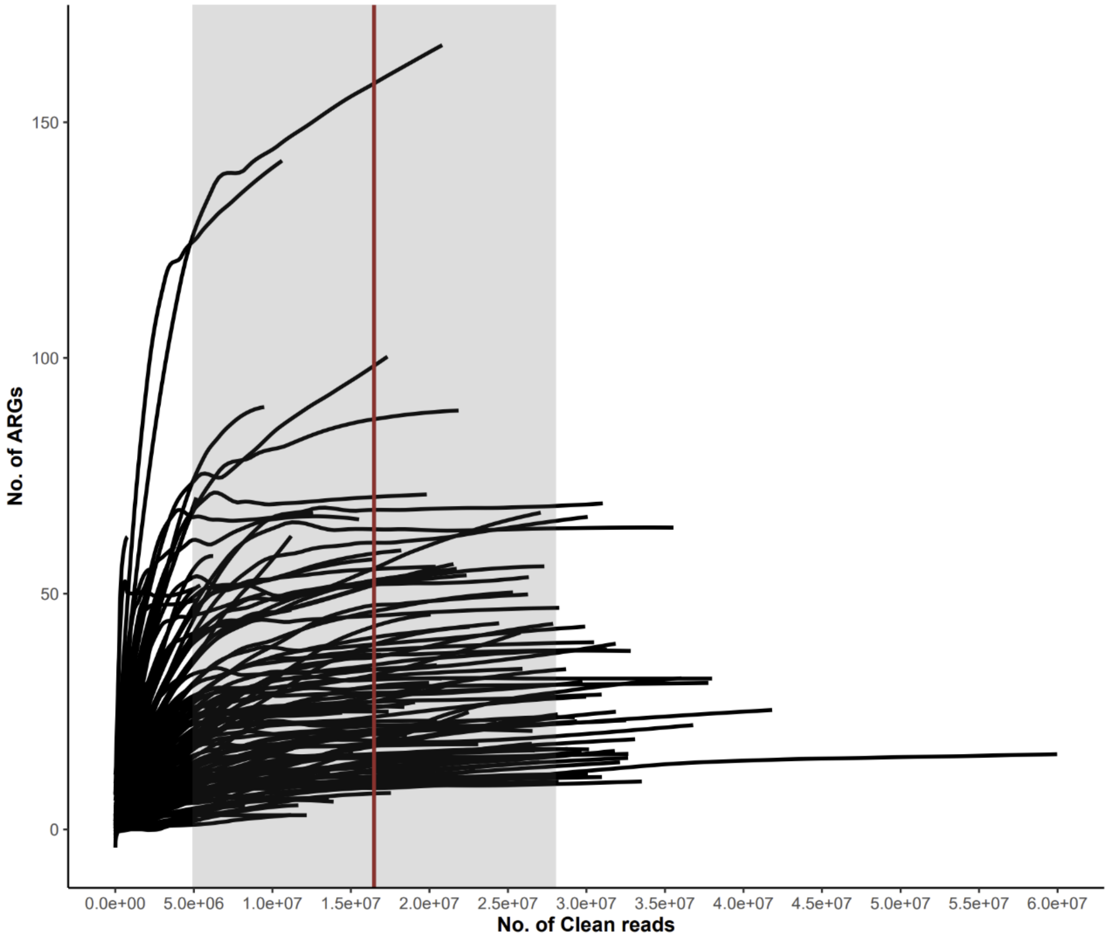

Supplementary Fig. 3: Rarefaction curve for the number of unique ARGs identified at subsampled sequencing depth. The red line indicates the median sequencing depth for all samples, while the gray shading represents one standard deviation. Source data are provided as a Source Data file.

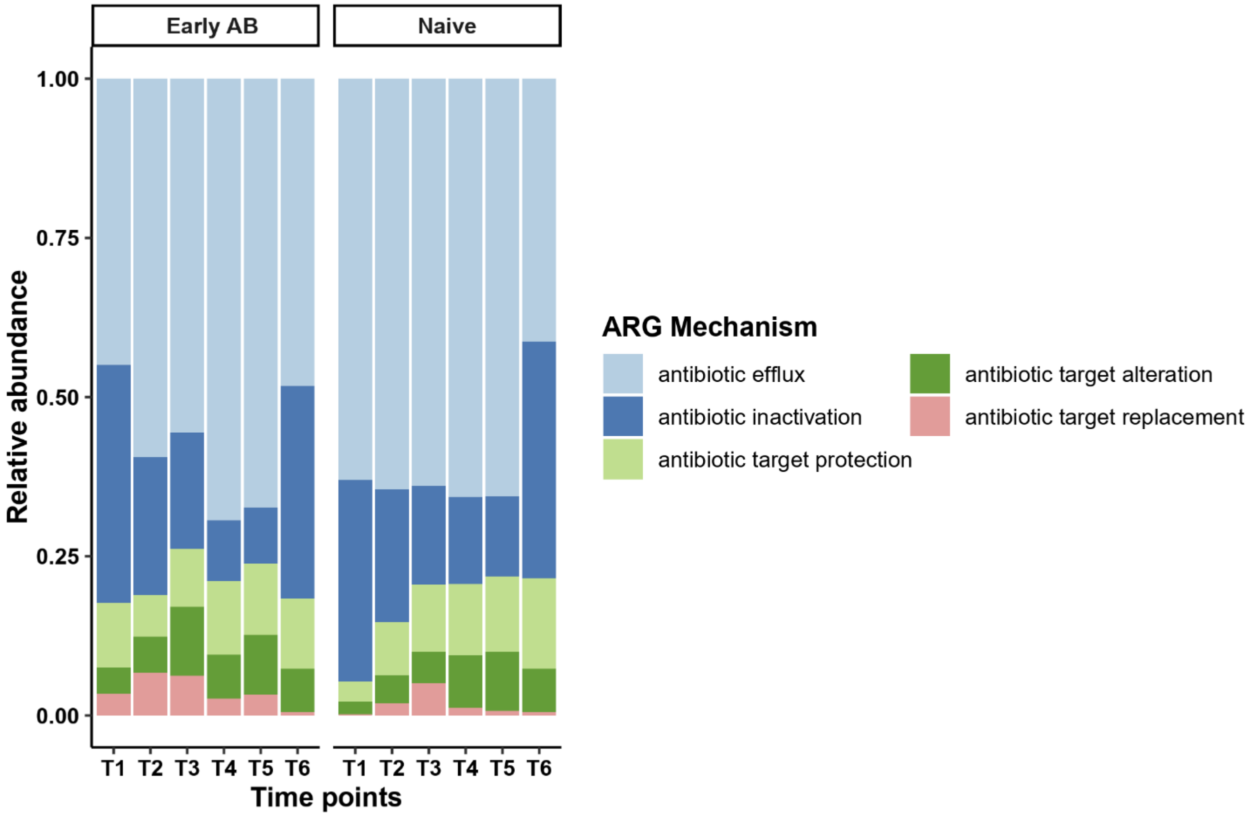

Supplementary Fig. 4: Mean normalized relative abundances of different ARG mechanisms found in the nasopharynx of early antibiotic-treated and naive preterm infants at different time points (T1-T6). Source data are provided as a Source Data file.

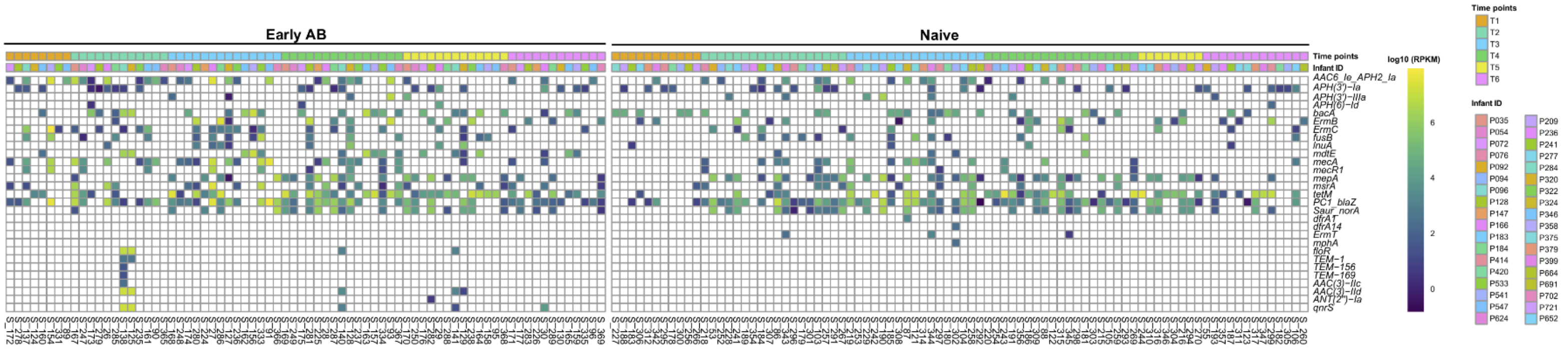

Supplementary Fig. 5: Heatmap showing the log10-transformed RPKM abundance of potential high-risk antibiotic resistance genes (ARGs) identified in the nasopharynx of all preterm infants across samples. The x-axis represents the names of samples, which are arranged by treatment group and time points. RPKM = reads per kilobase of reference gene per million bacterial reads. Source data are provided as a Source Data file.

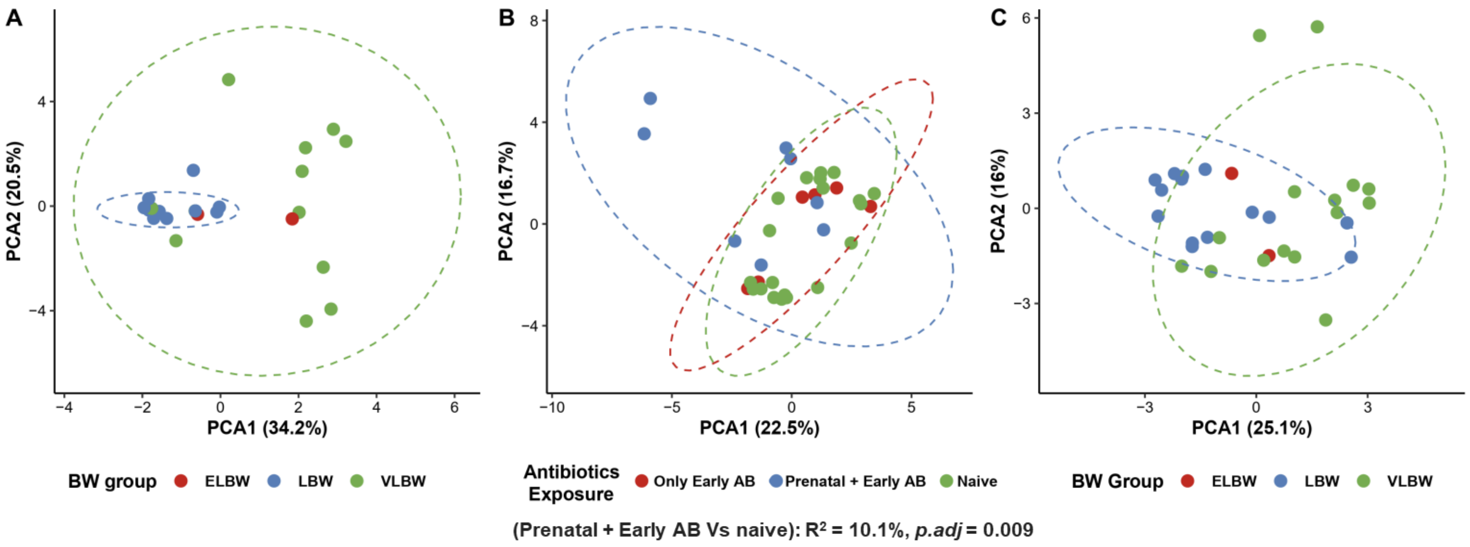

Supplementary Fig. 6: PCA visualizations of beta diversity analysis using the Aitchison distances at time points: (A) T1, (B) T2 and (C) T6 (6 month corrected age). Each point corresponds to a sample colored based on birth weight group (A, C) and prenatal and postnatal antibiotic exposure (B). Ellipses indicate 95% confidence intervals (CI). Adjusted P-values (corrected using the Benjamini–Hochberg method) and effect size (R^2) are based on PERMANOVA test. CLR = centered-log ratio; BW= Birth Weight. Source data are provided as a Source Data file.

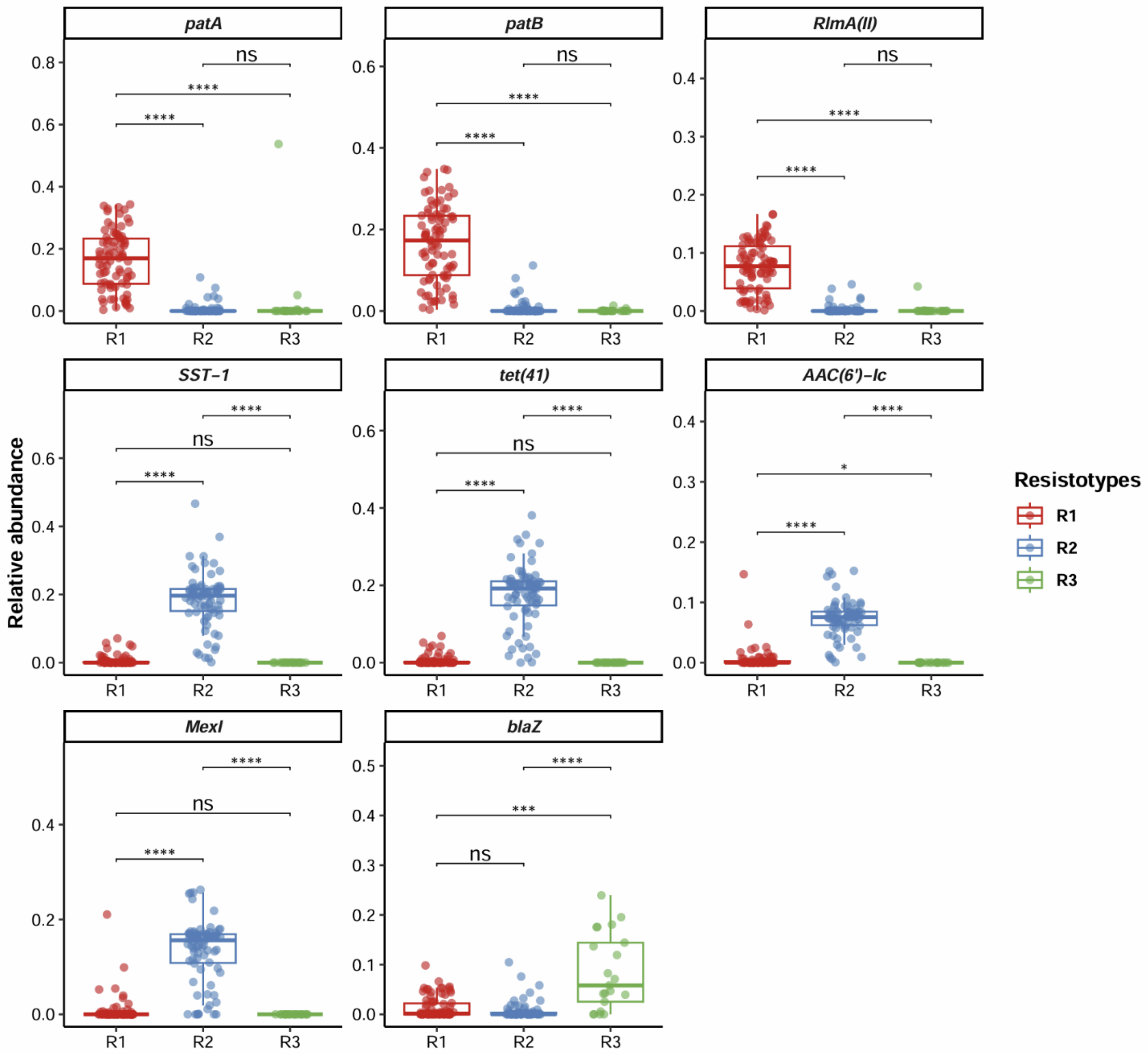

Supplementary Fig. 7: Box plots of the relative abundances of the main ARG contributors are shown for each Resistotype cluster identified across all the preterm infants' nasopharyngeal resistomes using DMM models. The X-axis displays the three identified clusters, and the Y-axis represents the relative abundance of the ARGs. DMM = dirichlet multinomial mixture; ns = not statistically significant. The statistical significance for the difference in the relative abundances of each ARGs in each Enterotype cluster is calculated using the two-sided pairwise Wilcoxon rank-sum test with Benjamini-Hochberg (BH) correction. Statistically significant differences are denoted with asterisk. *$p < .05$, **$p < .01$, ***$p < .001$ and ****$p < .0001$. Source data are provided as a Source Data file.

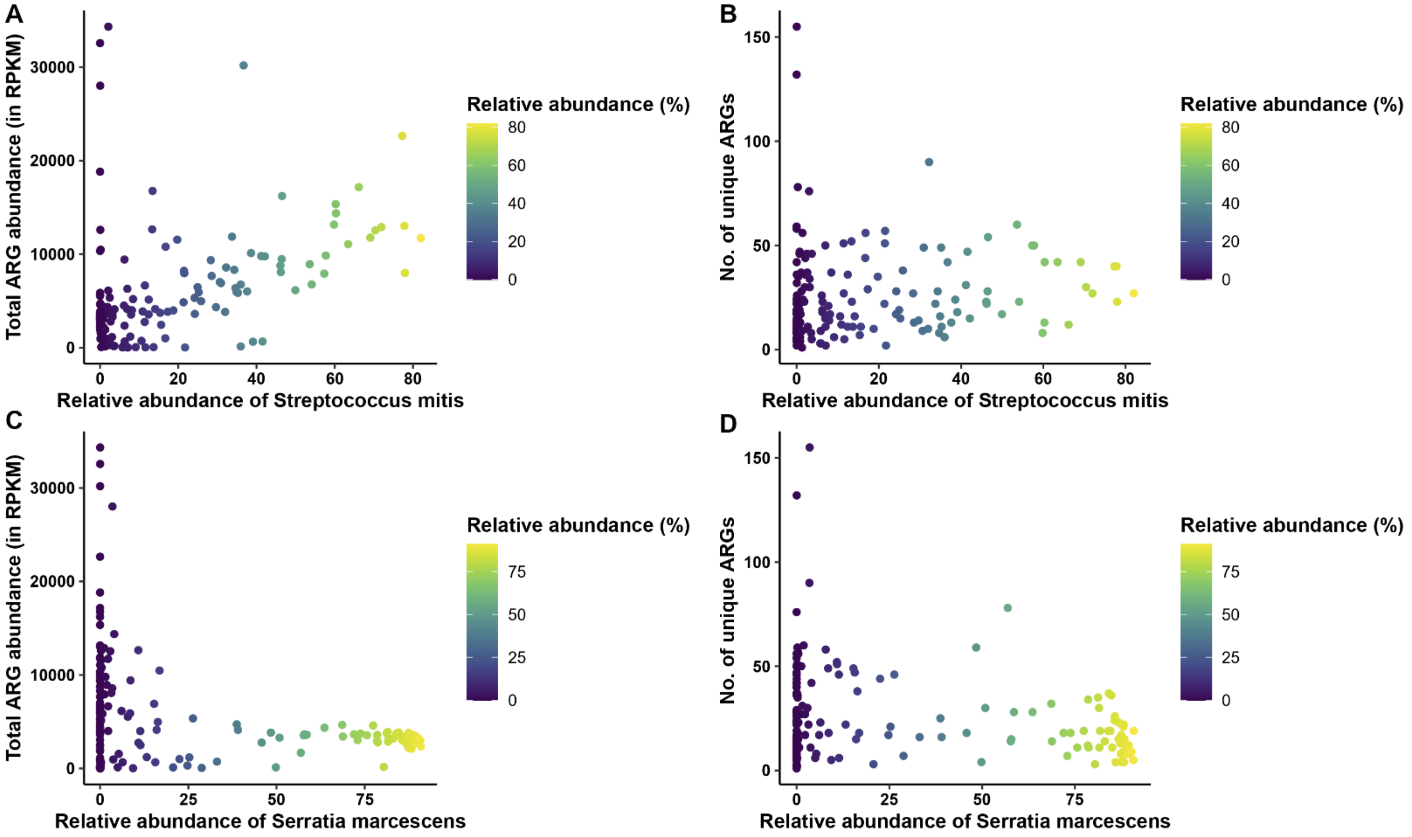

Supplementary Fig. 8: Scatterplots showing the association between correlated species and overall resistome outcomes across all infant samples from birth to 6 months corrected age. The total ARG abundance in RPKM was positively correlated with the relative abundance of *Streptococcus mitis* (A) and non-linearly negatively associated with the relative abundance of *Serratia marcescens* (C). Plots (B) and (D) depict the relationship between the number of unique ARGs and the relative abundance of *Streptococcus mitis* and *Serratia marcescens*, respectively. Plot (A) and (C) are colored by the relative abundance of *Streptococcus mitis*, while plots (B) and (D) are colored by the relative abundance of *Serratia marcescens*. Repeated measures correlation coefficients (r_{rm}) and adjusted P-values (adjusted using the Benjamini–Hochberg method) of the correlation were evaluated using repeated measures correlation (rmcorr) test. RPKM = reads per kilobase of reference gene per million bacterial reads. Source data are provided as a Source Data file.

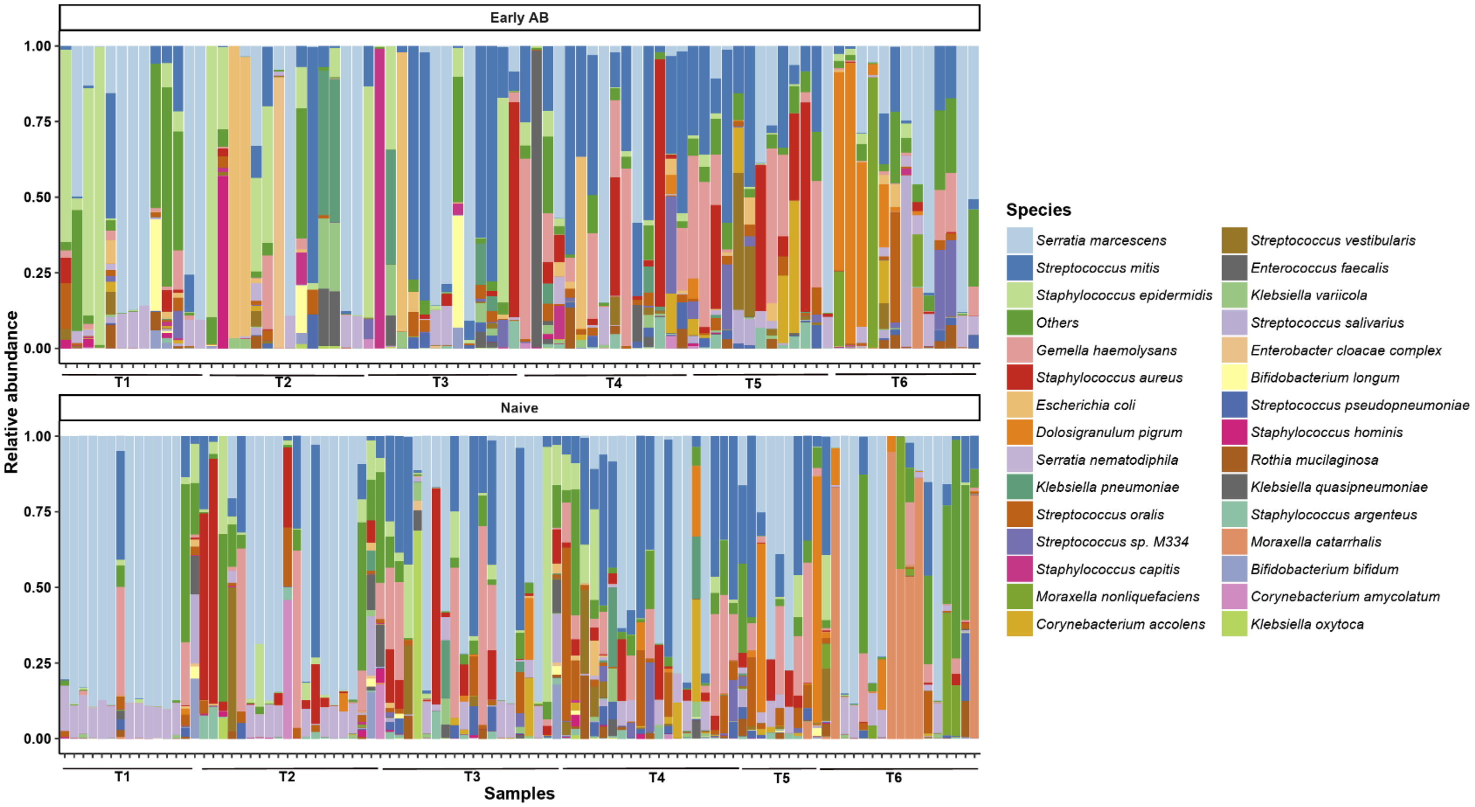

Supplementary Fig. 9: Stacked bar plots showing the relative abundance of top 30 most abundant bacterial species identified across samples in the antibiotic-treated (top) and naive (bottom) groups over sampling time points (T1-T6). All other low abundant species are merged into the "Others" category.

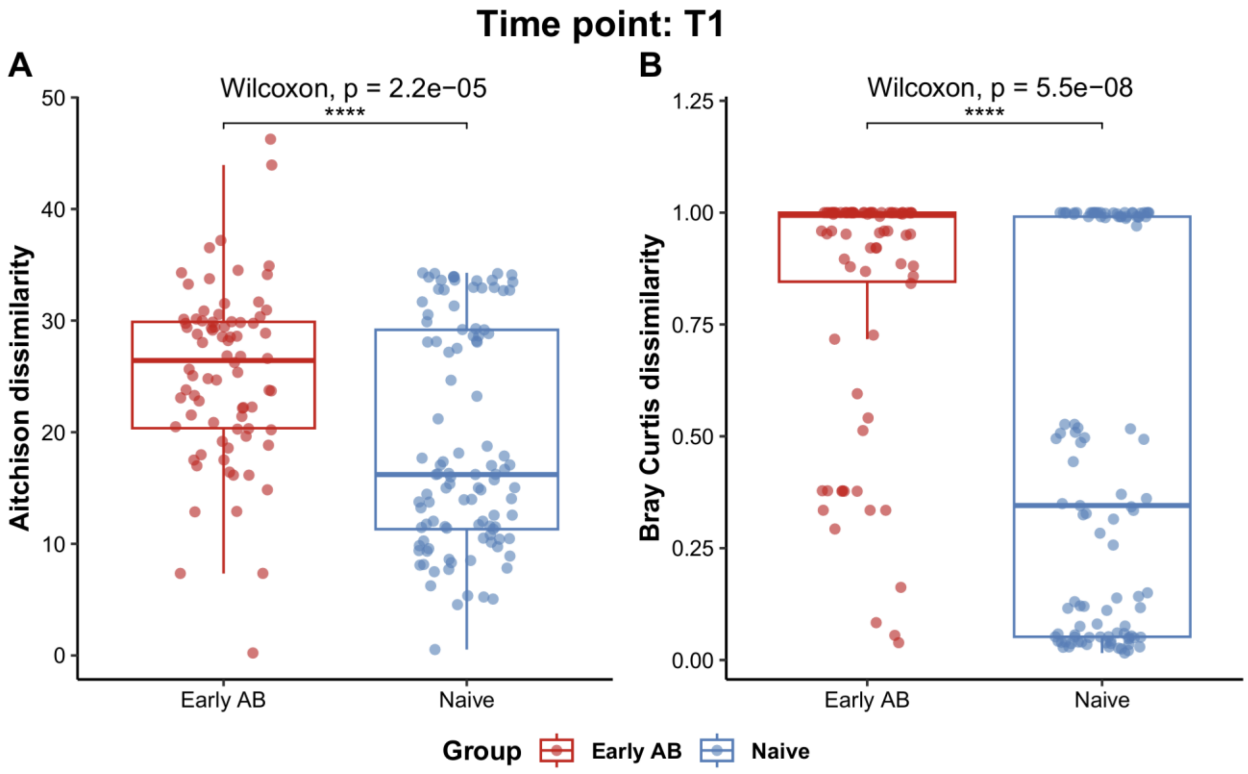

Supplementary Fig. 10: Boxplots comparing between-subject resistome dissimilarity measured using Aitchison (A) and Bray Curtis (B) distance in infants exposed to early antibiotics versus those without early antibiotic exposure (naive) at baseline (T1). P-values were generated by the two-sided Wilcoxon rank-sum test. $*p < .05$, $**p < .01$, $***p < .001$ and $****p < .0001$.

Supplementary Table 1

ALL infants (*n* = 36)

Time points	T1	T2	T3	T4	T5	T6	Total
Days of life (DOL)	0-3	4-9	12-19	20-31	32-76	229-288	
Extracted samples (before library prep)	35	37	36	35	23	32	198
Excluded samples (Low DNA yield)	2	1	1	0	0	0	4
Excluded samples (Low DNA yield) (%)	5.7%	2.7%	2.8%	0%	0%	0%	2%
Sequenced samples	33	36	35	35	22	32	194
Samples failed during Resistome profiling	5	1	2	0	1	1	10
Samples with double sampling time points	0	1	0	1	1	0	3
Included in the final analysis	28	34	33	34	21	31	181

Early AB infants (*n* = 15)

Timepoints	T1	T2	T3	T4	T5	T6	Total
Days of life (DOL)	0-3	4-9	12-19	20-31	32-76	229-288	
Extracted samples (before library prep)	15	16	15	16	14	14	90
Exclude samples (Low DNA yield)	0	0	1	0	0	0	1
Exclude samples (Low DNA yield) (%)	0%	0%	6.6%	0%	0%	0%	1.1%
Sequenced samples	15	16	14	16	14	14	89
Samples failed during Resistome profiling	2	1	0	0	0	1	4
Samples with double sampling timepoints	0	1	0	1	1	0	3
Included in the final analysis	13	14	14	15	13	13	82

Naive (*n* = 21)

Timepoints	T1	T2	T3	T4	T5	T6	Total
Days of life (DOL)	0-3	4-9	12-19	20-31	32-76	229-288	
Extracted samples (Before library prep)	20	21	21	19	9	18	108
Exclude samples (Low DNA yield)	2	1	0	0	0	0	3
Exclude samples (Low DNA yield) (%)	10%	5%	0%	0%	0%	0%	3%
Sequenced samples	18	20	21	19	9	18	105
Samples failed during Resistome profiling	3	0	2	0	1	0	6
Samples with double sampling timepoints	0	0	0	0	0	0	0
Included in the final analysis	15	20	19	19	8	18	99

Supplementary Table 1. Sample flowchart. Flowchart showing number of nasopharyngeal aspirate samples from preterm infants available at several steps in the study including at sampling, DNA extraction, sequencing and bioinformatics analysis.
