# Supplementary material for: Prolonged hospitalization signature and early antibiotic effects on the nasopharyngeal resistome in preterm infants
Source: Nat Commun. 2024 Jul 17;15:6024. doi: 10.1038/s41467-024-50433-7 (PMC11255206; doi:10.1038/s41467-024-50433-7)
Supplement: Supplementary file 3 — Description Of Additional Supplementary File [file 41467_2024_50433_MOESM3_ESM.pdf]

Description of Additional supplementary files

Supplementary Data 1: Sequence reads statistics.

Supplementary Data 2: Clinical covariates associated with the overall nasopharyngeal resistome composition.

Supplementary Data 3: DNA quantification data

Supplementary Data 4: Taxonomic profiles characterized at species-level using MetaPhlAn3.

Supplementary Data 5: Decontam result tables.
