# Supplementary material for: Prolonged hospitalization signature and early antibiotic effects on the nasopharyngeal resistome in preterm infants
Source: Nat Commun. 2024 Jul 17;15:6024. doi: 10.1038/s41467-024-50433-7 (PMC11255206; doi:10.1038/s41467-024-50433-7)
Supplement: Supplementary file 9 — Reporting summary [file 41467_2024_50433_MOESM9_ESM.pdf]

nature portfolio

Corresponding author(s): Fernanda Cristina Petersen

Last updated by author(s): Mar 28, 2024

Reporting Summary

Nature Portfolio wishes to improve the reproducibility of the work that we publish. This form provides structure for consistency and transparency in reporting. For further information on Nature Portfolio policies, see our Editorial Policies and the Editorial Policy Checklist.

Statistics

For all statistical analyses, confirm that the following items are present in the figure legend, table legend, main text, or Methods section.

n/a	Confirmed	
☐	☒	The exact sample size (*n*) for each experimental group/condition, given as a discrete number and unit of measurement
☐	☒	A statement on whether measurements were taken from distinct samples or whether the same sample was measured repeatedly
☐	☒	The statistical test(s) used AND whether they are one- or two-sided<br>*Only common tests should be described solely by name; describe more complex techniques in the Methods section.*
☐	☒	A description of all covariates tested
☐	☒	A description of any assumptions or corrections, such as tests of normality and adjustment for multiple comparisons
☐	☒	A full description of the statistical parameters including central tendency (e.g. means) or other basic estimates (e.g. regression coefficient) AND variation (e.g. standard deviation) or associated estimates of uncertainty (e.g. confidence intervals)
☐	☒	For null hypothesis testing, the test statistic (e.g. *F*, *t*, *r*) with confidence intervals, effect sizes, degrees of freedom and *P* value noted<br>*Give P values as exact values whenever suitable.*
☒	☐	For Bayesian analysis, information on the choice of priors and Markov chain Monte Carlo settings
☒	☐	For hierarchical and complex designs, identification of the appropriate level for tests and full reporting of outcomes
☐	☒	Estimates of effect sizes (e.g. Cohen's *d*, Pearson's *r*), indicating how they were calculated

Our web collection on statistics for biologists contains articles on many of the points above.

Software and code

Policy information about availability of computer code

Data collection

No software was used for the data collection.

Data analysis

All of the applied software is cited in the Methods section. We list the tools here:
- Trim Galore (v.0.6.1) : quality filtering and adapter trimming
- Bowtie2 (v.2.3.4.2): alignment of metagenomic reads against reference sequences
- SAMtools (v.1.9) and BEDTools (v.2.27.1): mapping reads and quantifying genes
- FastQC (v.0.11.9) and MultiQC (v.1.7): quality assessment of short reads.
- MetaPhlAn 3.0: metagenome taxonomic profiling
- Rarefaction Analyzer: rarefaction analysis
- R (v.4.2.1): statistical analysis and data visualization
- RStudio (v.2022.07.2+576): GUI for R
- R packages: decontam (v.1.16.0), phyloseq (v.1.40.0), ggplot2 (v.3.4.0), vegan (v.2.6.2), DirichletMultinomial (v.1.38.0), microbiome (v.1.18.0), rmcorr (v.0.5.4), pheatmap (v.1.0.12)
- Adobe Illustrator (v.16.0.0): figures editing

For manuscripts utilizing custom algorithms or software that are central to the research but not yet described in published literature, software must be made available to editors and reviewers. We strongly encourage code deposition in a community repository (e.g. GitHub). See the Nature Portfolio guidelines for submitting code & software for further information.

Data

Policy information about availability of data

All manuscripts must include a data availability statement. This statement should provide the following information, where applicable:

- Accession codes, unique identifiers, or web links for publicly available datasets
- A description of any restrictions on data availability
- For clinical datasets or third party data, please ensure that the statement adheres to our policy

Shotgun metagenomic sequencing reads generated during this study have been deposited in the NCBI Sequence Read Archive (SRA) database with BioProject ID PRJNA1009231. For review: (https://dataview.ncbi.nlm.nih.gov/object/PRJNA1009231?reviewer=811m19u88kg6no8nnf2eafjk99). The authors declare that all other data supporting the findings of this study (including source data for all figures) are available within the article, provided in the supplementary information files.
Databases used to analyse metagenomic sequencing data included:
1. Comprehensive Antibiotic Resistance Database (CARD) (https://card.mcmaster.ca/): for resistome profiling
2. MetaPhlan3 database (https://huttenhower.sph.harvard.edu/metaphlan3/): for microbiome profiling

Research involving human participants, their data, or biological material

Policy information about studies with human participants or human data. See also policy information about sex, gender (identity/presentation), and sexual orientation and race, ethnicity and racism.

Reporting on sex and gender	The findings are applicable to both sexes. Subjects were enrolled without regard to sex and gender, proportional to neonatal patient populations at Ullevål Neonatal Intensive Care Unit at Oslo University Hospital. While information on sex, rather than gender, was collected for all participants in the cohort at birth in accordance with ethical guidelines, it was not utilized in the study's analysis. The main aim of the study is to characterize the nasopharyngeal resistome in preterm infants, irrespective of infants sex.
Reporting on race, ethnicity, or other socially relevant groupings	Subjects were enrolled without regard to race, gender, or ethnic statuses, proportional to neonatal patient populations at Ullevål Neonatal Intensive Care Unit at Oslo University Hospital, Norway.
Population characteristics	21 antibiotic naive and15 early antibiotic treated preterm infants; Male: 23, Female:13; C-section: 28, Vaginal: 8. The characteristics of the study population are further detailed in Table 1.
Recruitment	We approached parents of all preterm infants with Gestational Age (GA) from 28 weeks 0 days to 31 weeks 6 days born at or transferred to Ullevål Neonatal Intensive Care Unit (NICU) at Oslo University Hospital, Norway, within 48 hours after birth between July 2019 and January 2021. Written informed consent was obtained from the infant's parents.
Ethics oversight	The study was performed in accordance with the Declaration of Helsinki and approved by the Hospital's Data Protection Officer and the Regional Committee for Medical and Health Research Ethics - South East, Norway (2018/1381 REKD).

Note that full information on the approval of the study protocol must also be provided in the manuscript.

Field-specific reporting

Please select the one below that is the best fit for your research. If you are not sure, read the appropriate sections before making your selection.

☒ Life sciences ☐ Behavioural & social sciences ☐ Ecological, evolutionary & environmental sciences

For a reference copy of the document with all sections, see nature.com/documents/nr-reporting-summary-flat.pdf

Life sciences study design

All studies must disclose on these points even when the disclosure is negative.

Sample size	No prior power calculations were performed, as calculating sample size for a longitudinal study with repeated measures is complex and requires simulation methods based on pilot datasets. The rationale for determining these sample sizes was based on an assessment of current literature and using similar sample numbers compared to other nasopharyngeal microbiome studies.
Data exclusions	To investigate temporal changes in the resistome in response to early antibiotics and control for potential baseline differences as confounding factors, this paper included only infants who had nasopharyngeal samples collected on the same day as the initiation of early antibiotic treatment (n = 15/33) and antibiotic-naive preterm infants as controls (n = 21). Nasopharyngeal samples were excluded from library preparation if there was insufficient DNA yield (n = 4). 10 samples were excluded from downstream analysis as no reads were assigned to ARGs. In addition, 3 samples were excluded due to double sampling time point. Detailed information on sample exclusion and inclusion statistics at different steps from sampling to metagenomic sequencing is also described in Supplementary Table 1.
Replication	No replication was used on metagenomic data as this was an observational study.

Randomization	This work was a non-interventional observational study. Ethical concerns prevented us from randomizing infants into antibiotic treated or naive groups. To account for potential confounding variables, we incorporated fixed effects controls into our mixed models.
Blinding	This work was a non-interventional observational study in which the preterm infants were assigned to antibiotic or naive groups based on their care needs. Blinding was not applicable.

Reporting for specific materials, systems and methods

We require information from authors about some types of materials, experimental systems and methods used in many studies. Here, indicate whether each material, system or method listed is relevant to your study. If you are not sure if a list item applies to your research, read the appropriate section before selecting a response.

Materials & experimental systems

n/a	Involved in the study
☒	☐ Antibodies
☒	☐ Eukaryotic cell lines
☒	☐ Palaeontology and archaeology
☒	☐ Animals and other organisms
☒	☐ Clinical data
☒	☐ Dual use research of concern
☒	☐ Plants

Methods

n/a	Involved in the study
☒	☐ ChIP-seq
☒	☐ Flow cytometry
☒	☐ MRI-based neuroimaging

Plants

Seed stocks	Not applied to this study
Novel plant genotypes	Not applied to this study
Authentication	Not applied to this study
